# Stainless Steel Wire Mesh Supported Molecularly Imprinted Composite Membranes for Selective Separation of Ebracteolata Compound B from *Euphorbia fischeriana*

**DOI:** 10.3390/molecules24030565

**Published:** 2019-02-04

**Authors:** Yukun Ma, Haijun Wang, Mengyan Guo

**Affiliations:** 1 College of Pharmacy, Qiqihar Medical University, Qiqihar 161006, China; mayukun@qmu.edu.cn; 2 Department of National Immunization Program, Qiqihar Center for Disease Control and Prevention, Qiqihar 161006, China; qscdcjm@163.com

**Keywords:** stainless steel wire mesh, *Euphorbia fischeriana*, Ebracteolata Compound B, molecularly imprinted composite membranes, selective separation

## Abstract

Stainless steel wire mesh supported molecularly imprinted composite membranes for selective separation of Ebracteolata Compound B (ECB) were prepared based on surface polymerization using ECB separated from *Euphorbia fischeriana* as a template, acrylamide as a functional monomer, ethylene glycol dimethacrylate as a cross-linker, azodiisobutyronitrile as an initiator, and stainless steel wire mesh as support. Structure and purity of ECB were characterized by nuclear magenetic resonance (^1^H-NMR, ^13^C-NMR) and ultra high performance liquid chromatography (UHPLC). The molecularly imprinted composite membranes were characterized by Fourier transform infrared spectroscopy (FTIR) and scanning electron microscope (SEM). The membrane adsorbed on the ECB reached equilibrium about 30 min later, with a maximum adsorption amount of 3.39 μmol/cm^2^. Adsorption behavior between ECB and the molecularly imprinted composite membranes followed pseudo-second-order kinetics equation and Freundlich isotherm model. The molecularly imprinted composite membranes that could selectively identify and transport ECB in similar structures have a permeation rate of 38.71% to ECB. The ECB content in the permeation solution derived from the extract of *Euphorbia fischeriana* through the imprinted membrane was 87%. Overall, the obtained results demonstrated that an efficient approach with the molecularly imprinted composite membranes for selective separation of ECB from *Euphorbia fischeriana*.

## 1. Introduction

*Euphorbia fischeriana* is a perennial herbaceous plant of euphorbiaceae found mainly in the Northeast of China. The plant’s dried roots, named “Lang-du” in traditional Chinese medicine, have many pharmacological effects of eliminating phlegm, antitubercle, and anti-cancer [1,2,3,4,5]. The Ebracteolata Compound B, ECB (2,4-dihydroxy-6-methoxy-3-methyl acetophenone), from the acetophenone family, is of concern and in great demand in the field of medicine due to its obvious anti-tuberculosis activity [6,7,8,9,10,11]. However, the separation and purification of ECB molecules by silica gel column chromatography is less efficient because of the low content in *Euphorbia fischeriana* [12]. Therefore, it is of great necessity to find a straightforward yet novel strategy for effective sensitive recognition, selective separation, and purification of ECB molecules in complex compositions from *Euphorbia fischeriana*.

Recently molecularly imprinted polymers are prepared by creating a three-dimensional polymeric matrix around a template molecule. After the removal of the template, imprinted cavities with complementary shapes and functional groups remain. Hence, molecularly imprinted polymers have attracted considerable attention owing of their specific recognition, structural reservation, and the wide range of potential applications in separation, catalysis, drug delivery, chemical sensing, and environmental detection [13,14,15,16,17,18,19,20]. Various types of molecularly imprinted materials with large surface areas have been synthesized, especially with respect to molecularly imprinted polymer membranes being used as separation tools, which can selectively transport a target molecule from a mixture solution by permeating through the thin membrane [21,22,23,24,25]. Molecularly imprinted composite membranes with the ultrafiltration or microfiltration supports are thinner, more flexible, and higher flux compared with the other freestanding membranes [26,27,28,29,30,31]. Selecting an appropriate support and functionalizing the support surface are the keys to obtaining high-quality membrane properties [32]. Such membrane technology has been already applied in many fields such as food [22], pharmaceutics [23], and environment [28]. Nevertheless there are few applications in the extraction and separation of the active components in the natural medicines.

Molecularly imprinted composite membrane generally was the surface layer with molecular imprinting function by means of the techniques of coating, interface polycondensation, surface grafting, or surface polymerization, etc. based on the existing commercial membranes which were ultrafiltration or microfiltration such as cellulose acetate, polysulfone, or polyacrylonitrile [24,25]. However, many molecularly imprinted composite membranes grown on the surface of the traditional flexible polymer substrates were prone to crack and curl. Molecularly imprinted polymers coated on a stainless steel wire as a selective solid-phase microextraction fiber have been successfully applied for determination of the drug molecules in biological fluids and tablet [33]. In order to further improve the practicability of stainless steel wire as a rigid substrate for molecularly imprinted materials, herein, we report the first preparation and characterization of continuous stainless steel wire mesh (SSWM) supported molecularly imprinted composite membranes of high quality. In this work, SSWM in terms of its elasticity, stability, permeability, relatively low cost, and ease of manufacture were selected as supports [34,35] when the molecularly imprinted composite membranes for selective separation of ECB (ECB-MICMs) were prepared. SSWM is resistant to high temperature, has high physical strength, and is stable in acids, bases, and non-polar solvents. The hydroxyl groups on the surface of SSWM easily bonds with silanylated coupling reagent, accordingly the vinyl-terminated silanylated coupling reagent can be selected to modify SSWM, so that the SSWM is suitable for polymerization with functional monomers, template molecules, and crosslinkers to prepare molecularly imprinted composite membranes. The obtained ECB-MICMs were characterized by SEM, TGA, and FT-IR. Moreover, the adsorption, permeation, and reusability properties were investigated. This proposed technique was shown to be a reliable and effective selective separation and purification method of ECB from *Euphorbia fischeriana*. The selective separation and purification of ECB in the extract of *Euphorbia fischeriana* by molecularly imprinted polymer membrane has greatly improved the purification efficiency due to the large amount of organic reagents saved and the cost and environmental pollution greatly reduced.

## 2. Results and Discussion

### 2.1. Validation of UHPLC Method

UHPLC chromatogram is shown in Figure S1. The ratio of the peak area of the target molecular to the total peak area of the sample is the purity of the sample. So the purity of ECB is 97%, which can meet the requirements of standard products and subsequent experiments. The calibration curve for ECB is in the range 0.25–2.00 mmol∙L^−1^. The regression equation is Y = 513854.8X + 253.9 (*R*^2^ = 0.9994).

### 2.2. Preparation of ECB-MICMs

**Figure 1:**
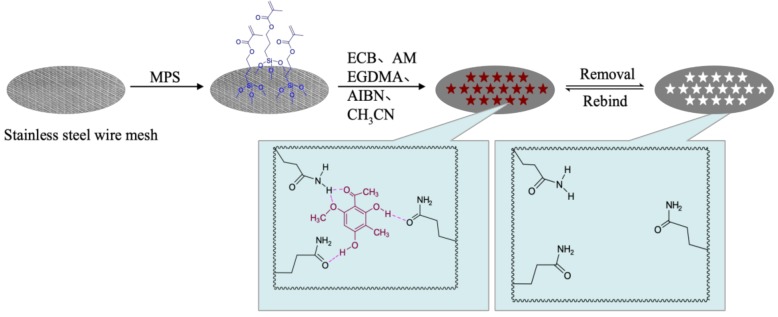
Schematic procedure for formation of a molecularly imprinted composite membrane for selective separation of Ebracteolata Compound B (ECB-MICMs) on a stainless steel wire mesh. MPS: 3-(methacryloyl) propyltrimethoxysilane; AIBN: azobisisobutyronitrile; EGDMA: ethylene glycol dimethacrylate AM: acrylamide.

The preparation process of the molecularly imprinted composite membranes is shown in Figure 1. The SSWM is an excellent rigid and flexile support to germinate membranes with the large flux. Firstly, the end vinyl was grafted onto the surface of SSWM using 3-(methacryloyl) propyltrimethoxysilane (MPS) as silanylated coupling agent. Subsequently, ECB and acrylamide were dispersed in acetonitrile for self-assemble, and then mixed with modified SSWM, crosslinkers, and initiators. Finally, a continuous molecularly imprinted composite membrane was synthesized on the surface of vinyl-modifed SSWM. The ECB were extracted from the molecularly imprinted composite membranes with solvent extraction, decomposing the hydrogen bond between ECB and acrylamide. The ECB-MICMs with molecular recognition ability were acquired owing to the imprinted cavities having excellent compatibility of size, shape, and chemical interactions.

### 2.3. Screening and Optimization of Functional Monomer

**Figure 2:**
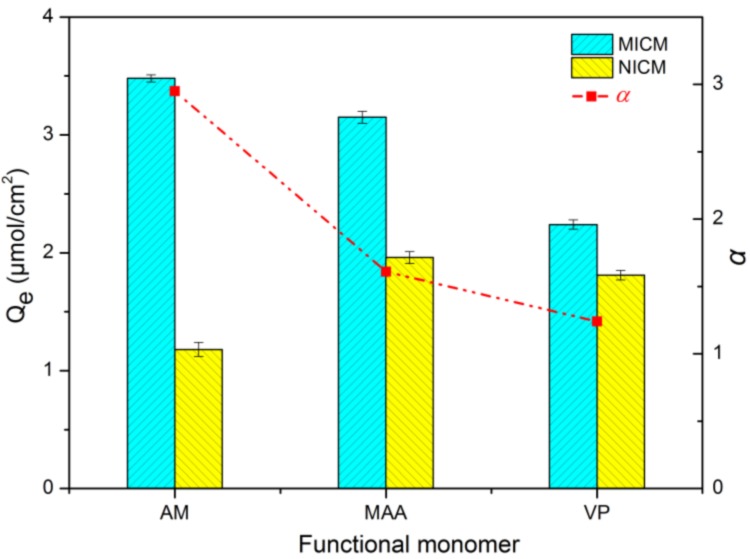
Effect of monomer species on adsorption capacity and imprinting factor of ECB.

Three commonly used functional monomers, acrylamide (AM), methacrylic acid (MAA), and 4-vinylpyridine (VP), were investigated in preparation of the ECB-MICMs. Effect of monomer species on the adsorption capacity and imprinting factor of ECB is shown in Figure 2. The adsorption capacity of the molecularly imprinted membrane prepared with acrylamide, methacrylic acid, and vinylpyridine as monomers to ECB decreased successively. The imprinting factors (α = Q_e, MICM_/Q_e, NICM_) of ECB on the membranes prepared using acrylamide, methacrylic acid, and vinylpyridine monomers were calculated as 2.95, 1.61, and 1.24, respectively. Therefore, acrylamide is the most suitable monomer owing its imprinting factor of ECB is obviously better than other monomers. The reason for the best imprinting efficiency may be that the force between acrylamide and ECB is stronger than that of other monomers due to more hydrogen bonds formed.

The dosage of functional monomer is determined by the structure of the template molecule. The ratio of the dosage of template molecule and functional monomer directly affects the specific recognition ability of the prepared molecularly imprinted composite membrane on target molecule. The amount of functional monomers in the polymerization system is too much, and the prepared molecularly imprinted composite membrane generated non-specific adsorption on the target, which reduced the imprinting factor of the molecularly imprinted composite membrane. On the contrary, the amount of functional monomers in the polymerization system is too small, the specific recognition cavities will be reduced. Therefore, the amount of functional monomer has a great influence on the specific adsorption capacity of the molecularly imprinted composite membrane.

**Figure 3:**
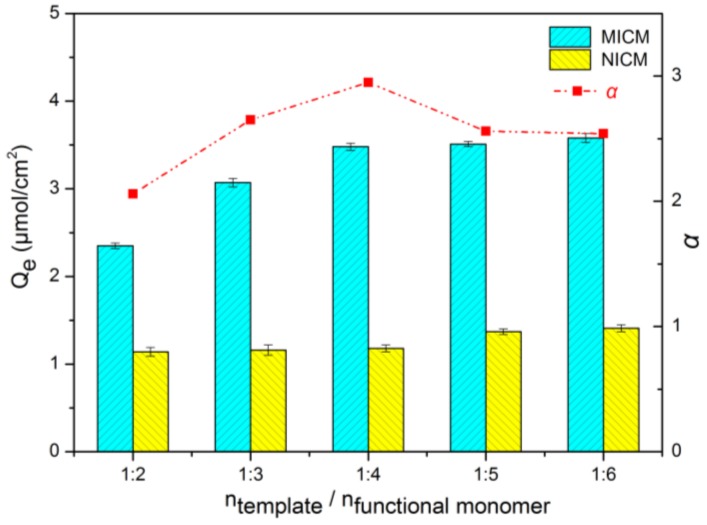
Effect of monomer content on adsorption capacity and imprinting factor of ECB. MICM: molecularly imprinted composite membrane; NICM: non-imprinted composite membrane.

The adsorption capacity and imprinting factor of molecularly imprinted composite membranes (MICMs) and non-imprinted composite membranes (NICMs) toward ECB with the different amounts of the functional monomers were investigated, and the results are shown in Figure 3. With the increase of acrylamide dosage, the trend of the adsorption amount of MICMs and NICMs to the target is increased gradually, and the imprinting factor is to increased first and then decreased. When the acrylamide concentration is too low, stable template-functional monomer complexes cannot be formed, resulting in fewer stable and effective imprinting sites formed with ECB. Therefore, imprinting efficiency is low and imprinting factor is small. As the amount of functional monomer increases, the equilibrium moves toward the direction of template-functional monomer complex. With the increase of the number of functional groups in the membranes, the adsorption amount of MICMs and NICMs to the target molecule ECB increases continuously. At the same time, the number of imprinting sites between acrylamide and ECB also accrued, and the imprinting factor enlarged. When the ratio of acrylamide to ECB is 5:1, the adsorption capacity of MICMs and NICMs to target molecules increases, but the imprinting factor decreases, which because the excessive acrylamide will lead to non-specific interaction between crosslinker–monomer and monomer–monomer, resulting in an increase in the non-selective binding sites. Consequently, ECB-MICMs prepared by ECB and acrylamide at a molar ratio of 1:4 is suitable.

### 2.4. Characterization of ECB-MICMs

**Figure 4:**
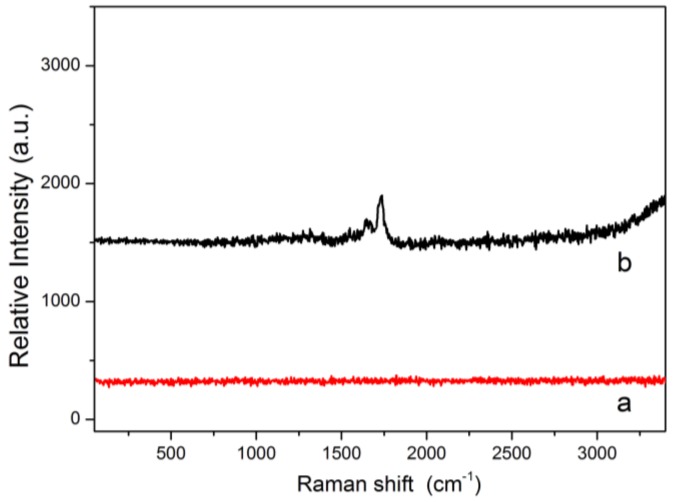
Raman spectra of the stainless steel wire mesh (**a**) and the MPS functionalized stainless steel wire mesh (**b**).

The Raman spectra of the stainless steel wire mesh and the MPS functionalized stainless steel wire mesh are shown in Figure 4. The stainless steel metal support has no Raman scattering characteristic peak, as shown in Figure 4a. The peaks between 1600 cm^−1^ and 1800 cm^−1^ in Figure 4b indicate that the surface structure of the stainless steel wire mesh has changed, which can be attributed to the Raman scattering characteristic peaks of C=O and R_2_C=CH_2_ in the MPS layer.

**Figure 5:**
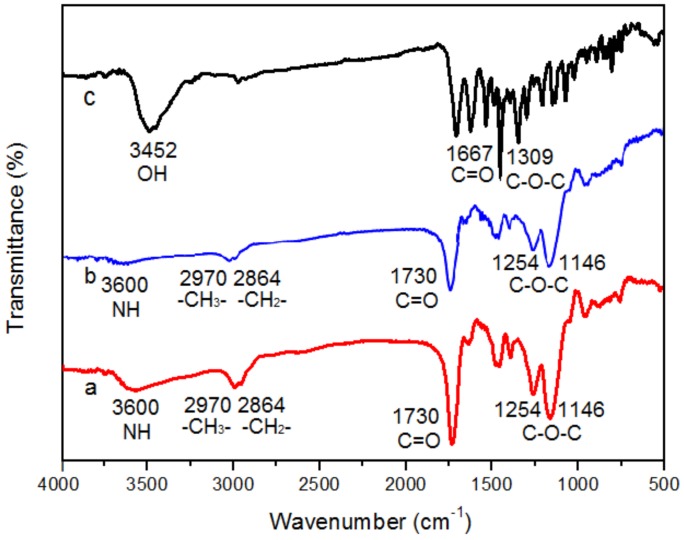
FT-IR spectra of ECB-MICMs (**a**), NICMs (**b**), and ECB (**c**).

The IR spectra of ECB-MICMs (a), NICMs (b), and ECB (c) are shown in Figure 5. The IR spectrum of 5c indicates that the stretching vibrations at 3452 cm^−1^, 1667 cm^−1^, 1309 cm^−1^ which correspond to –OH, C=O and C–O–C stretching vibrations, respectively. The adsorption peak at 2970 cm^−1^ and 2864 cm^−1^ in the spectra of 5a and 5b corresponds to –CH_3_ and –CH_2_– stretching vibrations. The peak at 3600 cm^−1^ observed for 5a and 5b corresponds to the characteristic adsorption of –N–H of acylamino. The characteristic peaks at 1730 cm^−1^ in the spectra of 5a and 5b attributed to the C=O stretching of ethylene glycol dimethacrylate (EGDMA) and acrylamide (AM). The stretching vibrations at 1254 cm^−1^ and 1146 cm^−1^ attributed to C–O–C in the obtained 5a and 5b could be observed.

**Figure 6:**
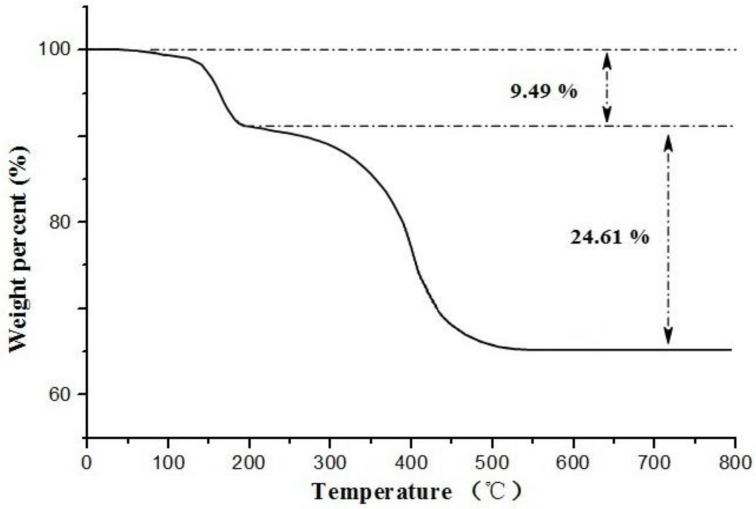
Thermo gravimetric analysis (TGA) curve of ECB-MICMs.

Figure 6 shows TGA curve of ECB-MICMs, the loss of weight below 150 °C can be ascribed to the loss of the residual or adsorbed solvent. Then the slight loss of weight occurs from 150 to 350 °C, which may be assigned to the decomposition and vaporization of various oxygen-containing functional groups. After that, the abrupt loss of weight occurs from 350 °C to 550 °C, which may be assigned to the degradation of carbon skeleton of MICMs. It is further suggested the successful synthesis of MICMs on the SSWM surface.

**Figure 7:**
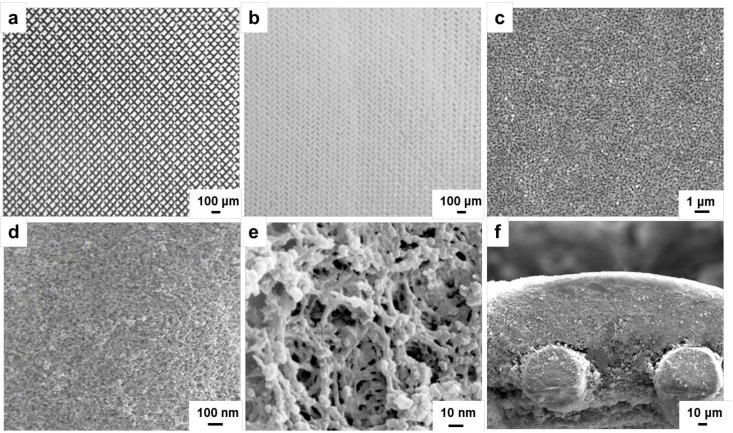
Optical micrographs of stainless steel wire mesh (SSWM) (**a**) and SSWM supported ECB-MICM (**b**). Scanning electron microscopy (SEM) of ECB-MICM (**c**–**e**) observed at different magnification, and the lateral (**f**) morphology.

A piece of continuous and uniform white membrane was grown on the SSWM surface with smooth and no cracks. Its morphology was characterized by optical microscope and scanning electron microscope. The morphology of the SSWM and the MICM under the optical microscope is shown in Figure 7a,b. The surface of the SSWM presents a certain metallic luster, and the size of the mesh hole is about 100 μm. The imprinted membrane has continuous symbiosis without obvious defects. Figure 7b–e show the morphology of the molecularly imprinted membrane observed at different magnification, and 7f shows the lateral morphology of the membrane. The thickness of stainless steel wire mesh supported molecularly imprinted composite membrane is around 100 μm. The porous reticular molecular imprinting membrane grows on the surface of the SSWM, with pores ranging from about 10 to 50 nanometers.

### 2.5. Adsorption of ECB on ECB-MICMs

#### 2.5.1. Adsorption Kinetics

**Figure 8:**
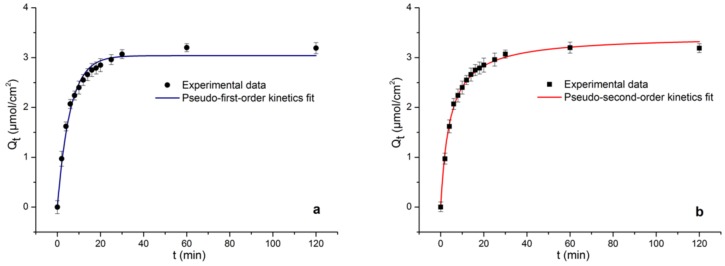
Fitted curve of Pseudo first order kinetic adsorption model (**a**) and fitted curve of Pseudo second order kinetic adsorption model (**b**) of ECB-MICMs.

Figure 8 shows the kinetics of ECB adsorption on MICMs. The adsorption amounts of ECB rapidly increases within 10 min, there are more recognition sites in the membrane structure during this period. And the increase trend slows down at 10~20 min, due to the recognition sites and cavities are mostly occupied by ECB molecules, until the adsorption equilibrium is reached at about 30 min. Pseudo-first-order and pseudo-second-order models are applied to fit the kinetic data. Figure 8a is the fitting curve of the first-order kinetic model (Q_t_ = Q_e_(1 − e^−kt^)); Figure 8b is the fitting curve of the second-order kinetic model (Q_t_ = kQ_e_^2^t/(1 + kQ_e_t)). Where Q_t_ is membrane adsorption amount to ECB when adsorption time is t, Q_e_ is equilibrium adsorption amount, k represents the rate constant, and t is the adsorption time, respectively.

Table 1 is the regression parameter of the fitting curve, in which the *R^2^* fitted by the pseudo-second-order kinetic model is closer to 1. The theoretical adsorption amount, Q_t_ = 3.4379 mmol∙cm^−2^, which is closer to the actual measurement data (3.27 mmol∙cm^−2^). Therefore, the second-order kinetic model is better to describe the adsorption process between ECB and MICMs.

#### 2.5.2. Adsorption Isotherms

**Figure 9:**
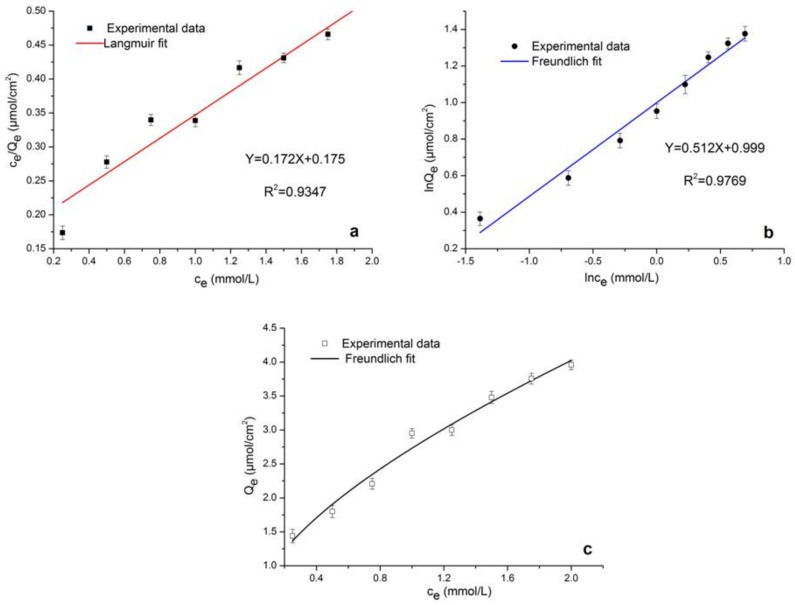
Linear fitted curve of Langmuir isothermal adsorption model (**a**), linear fitted curve of Freundlich isothermal adsorption model (**b**), and nonlinear fitted curve of Freundlich isothermal adsorption model (**c**) of ECB-MICMs.

Figure 9 displays the equilibrium adsorption of ECB on MICMs measured in different initial concentrations, which is the fitting curve of the isothermal adsorption model of the membrane to the ECB. The experimental data in Figure 9c shows that the adsorption capacities increased with the increase of initial concentrations, and the adsorption saturated when the concentration of ECB reached 3.96 mmol∙L^−1^. The Langmuir and Freundlich isotherm models are employed to describe the ECB sorption process. Figure 9a is the linear fitted curve of Langmuir isothermal adsorption model. Figure 9b is the linear fitted curve of Freundlich isothermal adsorption model. The calculated values for the correlation coefficient (*R^2^)* and the linear fitted equation are shown in Table 2. The correlation coefficient *R*^2^ of the plots indicates that the Freundlich isotherm model better describes the sorption of ECB on MICMs than the Langmuir isotherm model. The Freundlich isotherm model gives the relationship between equilibrium liquid and solid phase capacity based on multilayer adsorption. Figure 9c is the nonlinear fitted curve of Freundlich isothermal adsorption model.

### 2.6. Permeability of ECB-MICMs

**Figure 10:**
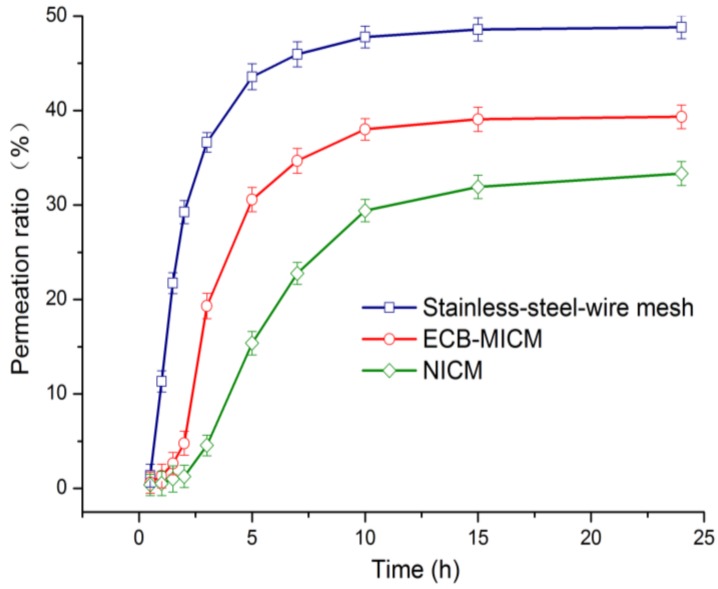
Permeability curves of SSWM and ECB-MICMs, and permeability device.

Figure 10 shows the permeability curves of SSWM, ECB-MICM, and NICM over time. The permeability curve of SSWM shows that the permeation balance can be achieved within 5 h, which is a high-flux membrane substrate with good permeability. The permeability curve of ECB-MICM is lower than that of the basement membrane, which further verifies the continuity of the imprinted membrane on the surface of the basement membrane. The permeability was basically balanced after 10 h at atmospheric pressure. The permeability curve of NICM was lower than that of MICM, and the permeability equilibrium time reached after 20 h was later than that of MICM, which is because NICM has a non-specific adsorption on ECB, and its adsorption capacity and permeability are both lower than MICM.

### 2.7. Permeation Selectivity

**Figure 11:**
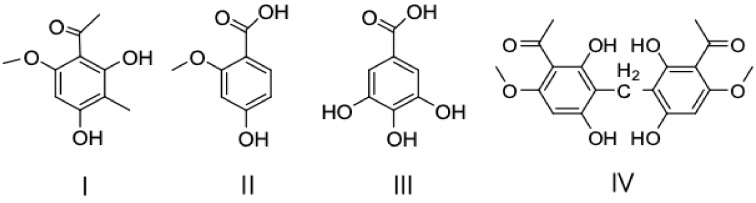
Structures of ECB (І) and three other similar compounds (II, III, and IV).

Permeation selectivity of the membrane was investigated by four research substrates, which were ECB (І) and three other similar compounds (II: 4-hydroxy-2-methoxybenzoic acid, III: 3,4,5-trihydroxybenzoic acid, and IV: 3,3′-diacetyl-4,4′-dimethoxy-2,2′,6,6′-tetrahydroxydiphenylmethane) isolated from *Euphorbia fischeriana*. The structures of the four substrates are shown in Figure 11.

**Figure 12:**
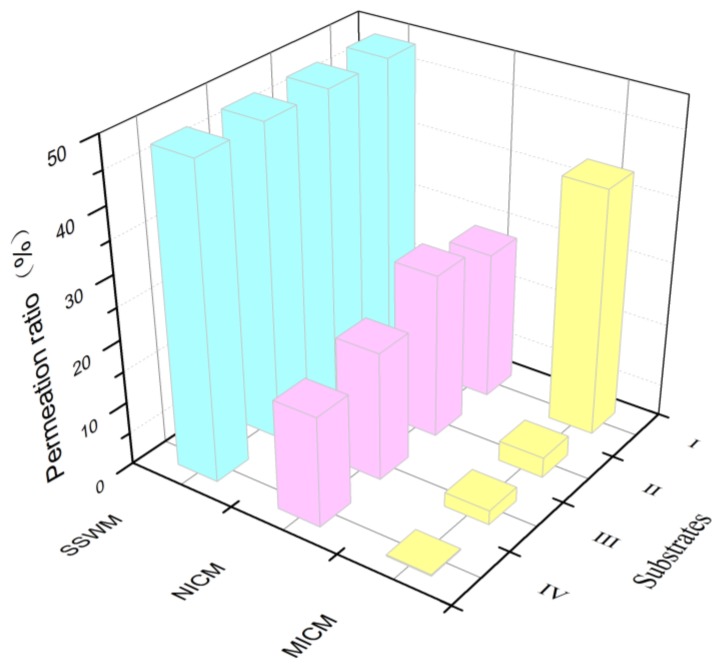
Permeation Selectivity of ECB-MICMs to ECB.

As shown in Figure 12, the permeation ratio of the MICM to the ECB (compound I) is significantly higher than that of compound II, III, and IV at the same permeation time. The template molecules are identified preferentially and absorbed sequentially by the cavities due to their matching with the complementary cavities and recognition sites of the membrane. The competitors II, III, and IV also have the opportunity to form hydrogen bonds with the imprinting sites. Nevertheless, the shapes of these competitors are not complementary to the shapes of the membrane cavities. Alternatively, they are blocked by the template molecules in the cavities that have been preferentially adsorbed. Therefore, the ECB firstly are enriched on the membrane, and then driven by the concentration gradient, selectively transfers through the membrane channel from one side to the other side continuously until the dynamic equilibrium reached. By calculation, the permeation ratio of membranes to ECB reached 38.71%. However, the permeability of the SSWM to all four compounds is about 48%. This result proves that the SSWM is not selective for the four substances, but it is a perfect permeable support with large flux. In the same way, NICM showed no significant difference in the permeation capacity of compounds I, II, III, and IV, and the average permeation ratio to the four compounds are about 20%, indicating no ability to selectively permeate the target molecules.

### 2.8. Reusability of ECB-MICMs

**Figure 13:**
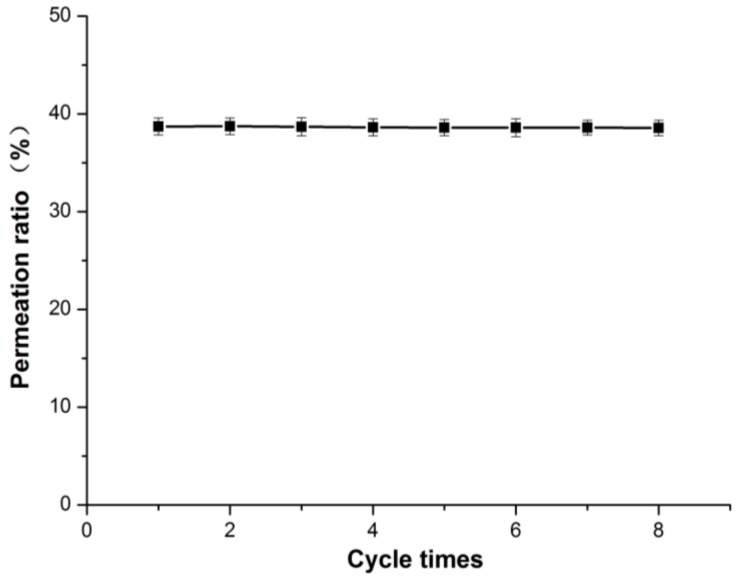
Reproducibility of ECB-MICMs.

The reuse ability is an important index of molecular imprinted materials. The reusability of the MICMs was evaluated by determining the permeability of the MICMs after regeneration. After the permeation selectivity experiment, the MICMs could be regenerated by repeated elution with methanol/acetic acid (9/1, *v/v*) until no ECB was detected, and then reused for the permeation of ECB. As shown in Figure 13, the MICMs had no significant change in the ECB’s permeability after 8 cycles, indicating that the membrane could be reused at least 8 times. This result implies that the MICMs are stable binding with support and can be recycled.

### 2.9. Separation of ECB in Euphorbia fischeriana Extract

**Figure 14:**
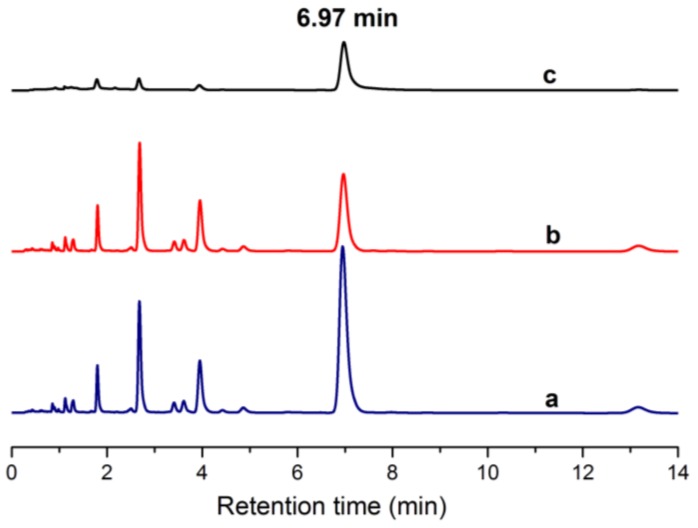
Ultra high performance liquid chromatography (UHPLC) chromatograms of the original solution of *Euphorbia fischeriana* extract (**a**), the residual solution of *Euphorbia fischeriana* extract (**b**) and the permeate solution through the membrane (**c**).

Figure 14 shows that the retention time of ECB peak area is 6.97 min. Figure 14b is the chromatogram of the residual solution of *Euphorbia fischeriana* extract after immersion for 10 h on the membrane. Figure 14a is the chromatogram of the original solution of *Euphorbia fischeriana* extract. The ECB’s peak area of chromatogram (b) is significantly lower than that of chromatogram (a). Figure 14c is the chromatogram of the permeate solution through the membrane, which shows that the ECB is very pure and its content is calculated to be 87%, with few impurities in the permeate solution. Finally, the ECB-MICMs showed a good selective permeation and separation of ECB from *Euphorbia fischeriana* extract.

## 3. Materials and Methods

### 3.1. Materials

*Euphorbia fischeriana* was supplied by Medicinal Botanical Garden of Qiqihar Medical University. Stainless steel wire mesh was purchased from Xinxiang Metal Network Co. (Henan, China). Ethylene glycol dimethacrylate (EGDMA), Azobisisobutyronitrile (AIBN), 3-(methacryloyl)propyl trimethoxysilane (MPS), and acrylamide (AM) were purchased from Energy-chemical (Shanghai, China). All other materials (piperidine, acetic acid, ethyl acetate, acetonitrile, petroleum ether, anhydrous methanol, and anhydrous ethanol) were purchased from Tianjin Fuyu Fine Chemical Co. (Tianjin, China). Silica gel (300–400 mesh) was used for chromatography.

### 3.2. Apparatus

^1^H and ^13^C-NMR spectra were measured on a Bruker Avance DRX600 spectrometer (Bruker, Billerica, MA, USA). Spot absorption spectra were obtained by use of a DXRxi Raman Imaging Microscope (Thermo Fisher Scientific, Waltham, MA, USA) with an excitation wavelength of 532 nm and 50 μm confocal pinhole diaphragm. Fourier transform infrared spectroscopy (FT-IR) was measured on a Bruker Equinox 55 Fourier transform infrared spectrometer (Bruker, Billerica, MA, USA). Scanning electron microscope (SEM) images were carried out using a HITACHI S-4300 scanning electron microscope (Hitachi, Tokyo, Japan). Thermo gravimetric analysis (TGA) was carried out with a Pyris-Diamond TGA (PerkinElmer, Boston, MA, USA). Ultra high performance liquid chromatography (UHPLC) was carried out with an American Waters Acquity UHPLC Class (Waters, Milford, MA, USA).

### 3.3. Experimental

#### 3.3.1. Extraction and Purification of ECB

*Euphorbia fischeriana* extract is extracted from heating the powder of *Euphorbia fischeriana* roots with alcohol before reflux, in which ECB was separated and purified by using silica gel column chromatography with gradientelution consisting of ethylacetate and petroleum ether. ^1^H-NMR (600 MHz, DMSO-*d*_6_), *δ* (ppm): 14.26 (1H, s, 2–OH), 6.06 (1H, s, H-5), 3.78 (3H, s, OCH_3_), 2.50 (3H, s, COCH_3_), 1.87 (3H, s, ArCH_3_), ^13^C-NMR (150 MHz, DMSO-*d*_6_), *δ* (ppm): 202.13, 164.52, 161.36, 104.22, 103.15, 91.24, 55.86, 32.97, 7.84. The NMR spectra are shown in Figures S3 and S4.

#### 3.3.2. Purity Measurement

The purity was determined by ultra-performance liquid chromatography. Chromatographic methanol dissolution samples, chromatographic conditions: The analysis was performed on Waters BEH C_18_ chromatographic column (2.1 mm × 50 mm, 1.7 μm). The mobile phase was acetonitrile-water (60/40, *v/v*), the flow rate was 0.4 mL∙min^−1^, and the detection wavelength was 290 nm. The purity calculated according to area normalization method.

The standard ECB were dissolved in methanol to a concentration of 10 mmol∙L^−1^. A series of standard solutions containing ECB were prepared at eight concentrations ranging from 0.25–2.00 µg∙mL^−1^. The standard curve of ECB was linear by assaying eight data points and measured three times.

#### 3.3.3. Preparation of ECB-MICMs

The SSWM was cut into a round piece with a diameter of 2 cm. The surface of SSWMs were polished by 800 mesh sandpaper, washed several times with deionized water and anhydrous ethanol, and dried in the 60 °C oven. Anhydrous ethanol/deionized water/MPS (90/6/4, *v/v*) was prepared into a 100 mL solution, which was adjusted to a pH of 4.5 by acetic acid (30 wt %). After stirring for 1 h, the solution was left to hydrolyze for 24 h. The SSWMs were placed in a completely hydrolyzed MPS solution without light and left out to dry after 24 h.

In order to non-covalently interact adequately between the template molecules and functional monomer, ECB (0.1 mmol), AM (0.4 mmol) were ultrasonic dissolved in acetonitrile (10 mL), and was placed in 4 °C refrigerator. After 6 h, took it out, mixed with EGDMA (2 mmol) and AIBN (0.1 mmol), injected with nitrogen for 15 min to remove oxygen, shook it by the water bath at 60 °C. Prepolymer solution was formed and removed after 12 h, in which the modified SSWM on the vertical support was fast placed. Polymerization takes place for 4 h at 60 °C in the vacuum drying oven. After being taken out at room temperature, the membrane surface was washed with deionized water and methanol. Then the membrane was eluted by methanol/acetic acid (9/1, *v/v*) until there was no ECB in the eluent. Non-imprinted composite membranes were prepared under the same conditions as imprinted membranes except without using ECB.

#### 3.3.4. Screening of Functional Monomer Experiment

Acrylamide, methacrylic acid, and vinylpyridine were selected as functional monomers to prepare molecularly imprinted and non-imprinted membranes according to the same experimental scheme as 3.3.3. The MICMs and NICMs were placed separately in the 1.0 mmol∙L^−1^ ECB standard solution (solvent was methanol) at room temperature for 10 h. The concentration of ECB was detected by UHPLC and the adsorption amount (Q_e_ = V (c_0_-c_t_)/A_m_, c_0_ was the initial concentration of ECB, c_t_ was the equilibrium concentration of ECB in the solution after a certain period of adsorption, V was the volume of the solution, A_m_ was the surface area of the membrane) was calculated.

#### 3.3.5. Optimization of Functional Monomer Experiment

MICMs prepared by ECB and acrylamide in molar ratios of 1:1, 1:2, 1:3, 1:4, and 1:5 were placed in the 1.0 mmol∙L^−1^ ECB standard solution at room temperature for 10 h. The adsorption capacity of the MICMs and the NICMs prepared in response to MICMs to ECB were measured.

#### 3.3.6. Characterization

The structure was characterized by infrared spectrum (the ECB and the scraped powder on the surface of ECB-MICMs, KBr compression tablets, 32 consecutive scans at a resolution of 2 cm^−1^, with a wavelength range of 4000~500 cm^−1^). The surface morphology was characterized by optical microscope and SEM. The thermodynamic stability of the membrane was characterized by thermo gravimetric analysis.

#### 3.3.7. Adsorption Experiment

ECB-MICMs were placed in the 1.0 mmol∙L^−1^ ECB standard solution, taken out at the appropriate time, the adsorption amount of ECB on the menbranes was determined by UHPLC (Q_t_ = V (c_0_-c_t_)/A_m_, c_0_ is the initial concentration of ECB, c_t_ is the equilibrium concentration of ECB in the solution after a certain period of adsorption, V is the volume of the solution, and A_m_ is the surface area of the membrane). The adsorption curve was fitted by the kinetic equation with the adsorption time (t/min) as the abscissa and the adsorption amount (Q_t_/μmol∙cm^−2^) as the ordinate.

ECB-MICMs were placed separately in the 0.25, 0.5, 0.75, 1.0, 1.25, 1.5, 1.75, and 2.0 mmol∙L^−1^ ECB standard solutions at room temperature for 10 h. ECB concentration was measured in turn by UHPLC to calculate the adsorption (Q_e_ = V (c_0_-c_t_)/A_m_, c_0_ was the initial concentration of ECB, c_t_ was the equilibrium concentration of ECB in the solution after a certain period of adsorption, V was the volume of the solution, A_m_ was the surface area of the membrane). The adsorption curve was fitted by isothermal model with Q_e_ (μmol∙cm^−2^) as the ordinate and c (mmol∙L^−1^) as the abscissa.

#### 3.3.8. Permeation Experiment

SSWM, ECB-MICM, and NICM were sequentially fixed between two connected containers as shown in Figure S2. The effective diameter of the SSWM and ECB-MICM was 2 cm. There was 80 mL of ECB (0.1 mmol∙L^−1^) methanol solution in one side, and there was the pure solvent with the same volume in the other side. With the magnetic stirring, the permeation amount of ECB was measured by UHPLC periodically and calculated the permeability (R_p_= Q_p_/Q_b_, Q_p_ is the permeation amount of ECB and Q_b_ is the initial amount of ECB).

#### 3.3.9. Permeation Selectivity Experiment

The membrane with an effective diameter of 2 cm was fixed on the permeation device. There was 80 mL of 1 mmol∙L^−1^ of the substrate methanol solution (the substrate was ECB, 3-methoxyl-p-hydroxybenzoic acid, gallic acid,3,3′-diacetyl-4,4′-dimethoxyl-2,2′,6,6′-tetrahydroxydiphenyl methane) in one side, and there was the pure solvent with the same volume in the other side. With magnetic stirring for 10 h, the permeation amount of substrate was measured by UHPLC and calculated the permeability (R_p_= Q_p_/Q_b_, Q_p_ is the permeation amount of substrate and Q_b_ is the initial amount of substrate).

#### 3.3.10. Reusability Experiment

The membrane was eluted with methanol/acetic acid (9/1, *v/v*) after the 3.3.9 experiment, and then repeated for 8 times according to the 3.3.9 experimental method, and the permeability of the membrane was detected respectively.

#### 3.3.11. Separation Performance of ECB-MICMs

The membrane with an effective diameter of 2 cm was fixed on the permeation device. There was 80 mL methanol solution containing the ECB of *Euphorbia fischeriana* extract in one side, and there was the pure solvent with the same volume in the other side. After 10 h of magnetic stirring, the residual solution of *Euphorbia fischeriana* extract, the permeate solution through the membrane and the permeable membrane were respectively taken out. The change of chromatogram peak area was observed by UHPLC.

## 4. Conclusions

In summary, the stainless steel wire mesh supported molecularly imprinted composite membranes for selective separation of ECB has been synthesized successfully by surface polymerization technology. A mechanism of membrane formation was proposed based on our experimental observations. The structure and quality of the membrane were ascertained from FTIR and SEM results. Specific adsorption behavior and selective permeability of molecularly imprinted composite membranes were studied systematically. The results showed that the adsorption equilibrium of the MICMs toward ECB was reached after about 30 min, with a maximum adsorption amount of 3.39 μmol/cm^2^. Adsorption behavior between ECB and the molecularly imprinted composite membrane followed pseudo-second-order kinetics equation and Freundlich isotherm model. The membrane, which had permeation rate of 38.71% to ECB, was a novel kind of separation material with special recognition ability. The ECB content in the permeation solution derived from the extract of *Euphorbia fischeriana* through the imprinted membrane was 87%. Thus, this technique can selectively separate the ECB from *Euphorbia fischeriana*, making the ECB separation efficiency significantly improved. It not only overcame the defect of complicated procedure and large amount of solvents in the traditional chromatography, but also avoided the troubles of centrifugation, filtration and reprocessing by the conventional molecular imprinting. In practical application, ECB can be achieved easily by permeating the extracts of *Euphorbia fischeriana* through this polymer membrane. The simple technique established for membrane formation offers an effective route to novel imprinted materials. Moreover, the stainless steel wire mesh could be used to produce other molecularly imprinted composite membranes.

## Figures and Tables

**Table 1:** Kinetic adsorption models constants of ECB-MICMs.

Kinetics Model	Parameters		
	*k*	Q_t_	*R^2^*
Pseudo-first-order	0.1691	3.03901	0.9860
Pseudo-second-order	0.0068	3.4379	0.9956

**Table 2:** Adsorption isotherm parameters of ECB-MICMs.

Isothermal Asorption Model	Parameters	
	Equation	*R^2^*
Langmuir	Y = 0.172X + 0.175	0.9347
Freundlich	Y = 0.512X + 0.999	0.9769

## Supplementary Materials

The following are available online, Figure S1: UHPLC chromatogram and calibration curve of ECB, Figure S2: The permeability device, Figure S3: The ^1^H-NMR Spectrum of compound ECB, Figure S4: The ^13^C-NMR Spectrum of compound ECB.
