# Supplementary material for: Stainless Steel Wire Mesh Supported Molecularly Imprinted Composite Membranes for Selective Separation of Ebracteolata Compound B from Euphorbia fischeriana
Source: Molecules. 2019 Feb 4;24(3):565. doi: 10.3390/molecules24030565 (PMC6384690; doi:10.3390/molecules24030565)
Supplement: Supplementary file 1 [file molecules-24-00565-s001.pdf]

Molecules **2018**

Supplementary material

Stainless Steel Wire Mesh Supported Molecularly Imprinted Composite Membranes for Selective Separation of Ebracteolata Compound B from *Euphorbia Fischeriana*

Yukun Ma [1], Haijun Wang [1,*] and Mengyan Guo [2]

1 College of Pharmacy, Qiqihar Medical University, Qiqihar 161006, China; kuntengchongtian@163.com;
2 Department of National Immunization Program, Qiqihar Center for Disease Control and Prevention, Qiqihar 161006, China; qscdcjm@163.com

* Correspondence: qmuhjwang@163.com; Tel.: +86-0452-2663-881

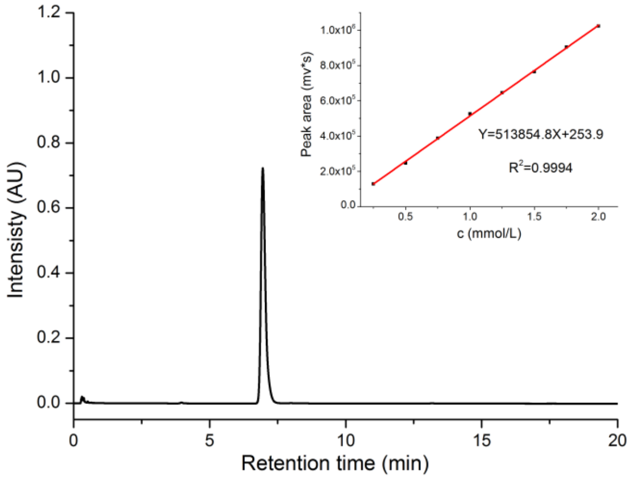

Figure S1. UHPLC chromatogram and calibration curve of ECB.

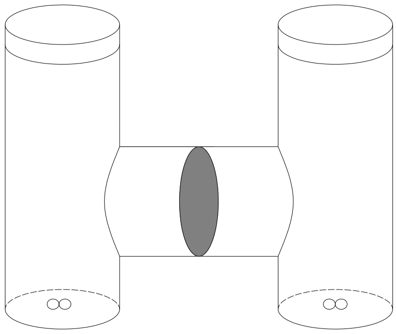

Figure S2. The permeability device.

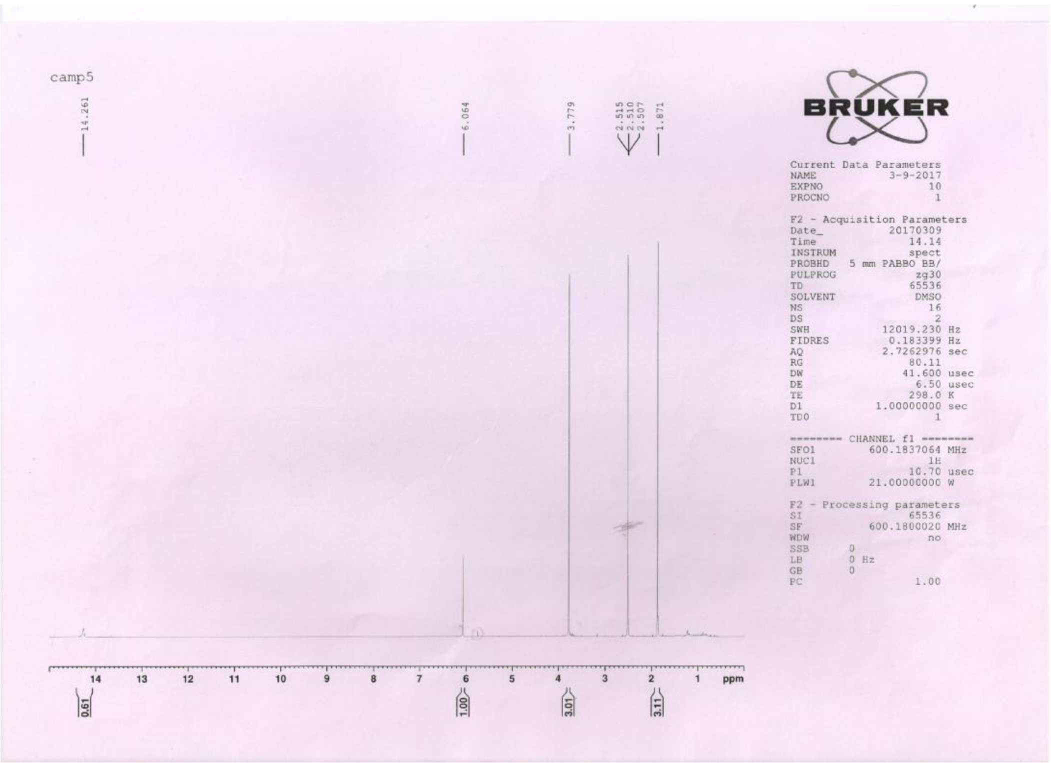

Figure S3. The ^{1}H-NMR Spectrum of compound ECB.

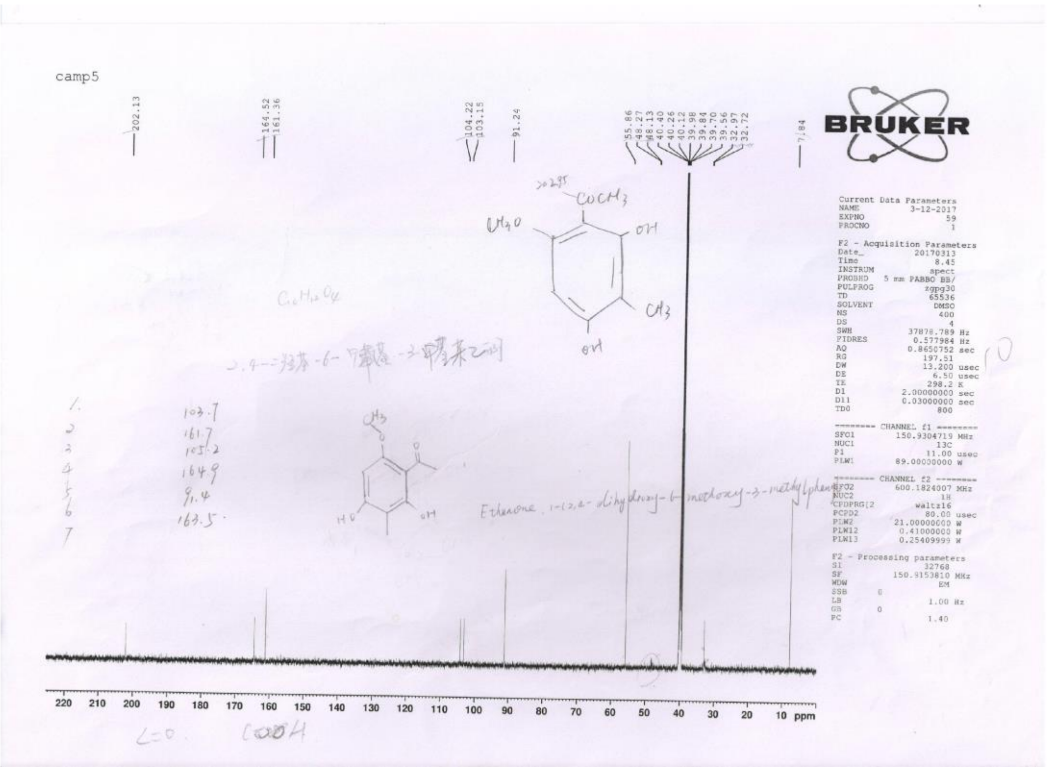

Figure S4. The ^{13}C -NMR Spectrum of compound ECB.
